# Endothelial structure contributes to heterogeneity in brain capillary diameter

**DOI:** 10.1530/VB-23-0010

**Published:** 2023-09-06

**Authors:** Sheridan M Sargent, Stephanie K Bonney, Yuandong Li, Stefan Stamenkovic, Marc M Takeno, Vanessa Coelho-Santos, Andy Y Shih

**Affiliations:** 1 Neuroscience Graduate Program, University of Washington, Seattle, Washington, USA; 2 Center for Developmental Biology and Regenerative Medicine, Seattle Children’s Research Institute, Seattle, Washington, USA; 3 Allen Institute for Brain Science, Seattle, Washington, USA; 4 Coimbra Institute for Biomedical Imaging and Translational Research, University of Coimbra, Portugal; 5 Institute of Nuclear Sciences Applied to Health, University of Coimbra, Portugal; 6 Department of Pediatrics, University of Washington, Seattle, Washington, USA; 7 Department of Bioengineering, University of Washington, Seattle, Washington, USA

**Keywords:** endothelium, imaging, microvasculature, vascular heterogeneity, vascular homeostasis

## Abstract

The high metabolic demand of brain tissue is supported by a constant supply of blood flow through dense microvascular networks. Capillaries are the smallest class of vessels in the brain and their lumens vary in diameter between ~2 and 5 μm. This diameter range plays a significant role in optimizing blood flow resistance, blood cell distribution, and oxygen extraction. The control of capillary diameter has largely been ascribed to pericyte contractility, but it remains unclear if the architecture of the endothelial wall also contributes to capillary diameter. Here, we use public, large-scale volume electron microscopy data from mouse cortex (MICrONS Explorer, Cortical mm^3^) to examine how endothelial cell number, endothelial cell thickness, and pericyte coverage relates to microvascular lumen size. We find that transitional vessels near the penetrating arteriole and ascending venule are composed of two to six interlocked endothelial cells, while the capillaries intervening these zones are composed of either one or two endothelial cells, with roughly equal proportions. The luminal area and diameter are on average slightly larger with capillary segments composed of two interlocked endothelial cells vs one endothelial cell. However, this difference is insufficient to explain the full range of capillary diameters seen* in vivo*. This suggests that both endothelial structure and other influences, including pericyte tone, contribute to the basal diameter and optimized perfusion of brain capillaries.

## Introduction

The brain is a highly active and metabolically demanding organ. Capillaries serve as the distribution network for oxygen-carrying blood cells and nutrient supply. Since the earliest *in vivo* imaging studies of the brain microcirculation, researchers have noticed a striking variance in blood flow and diameter among individual segments of capillaries (1, 2, 3, 4). Capillary segments of larger diameter tend to support higher blood flow, and these vessels are spatially intermingled with thinner capillaries supporting lower flow (5, 6). While capillary flow fluctuates on the timescale of seconds to minutes due to dynamic physiological processes such as vasomotion and neurovascular coupling (2, 7), these dynamics are built atop a relatively stable but heterogeneous pattern of capillary flow, set by capillary architecture and pericyte tone (6).

These findings raise the question of why heterogeneity in brain capillary flow is necessary. Recent studies suggest that heterogeneity creates reserve space for increased blood flow and tissue oxygenation during neural activity (i.e. functional hyperemia) (8). This is akin to the concept of capillary recruitment described in peripheral tissues, such as muscle, where inactive tissue contains a proportion of nonflowing or very low-flow capillaries, which can be recruited to flow during increased metabolic demand (9). However, capillaries with no (or very little) flow are not ideal for brain function given the energy demands of the brain, and there are mechanisms to recannulate capillaries after flow obstruction by circulating emboli (10, 11). The system therefore establishes a range of flow levels across all capillaries at baseline, where capillaries that completely lack flow are very rare. This baseline state of heterogeneity transitions to more homogeneous flow among capillaries segments during increased brain activity. That is, low flux capillaries increase in flow, and high flux capillaries slightly decrease in flow (12, 13). Flow homogenization promotes a more even distribution of oxygenated blood cells among capillaries within the network, and slows their transit time to maximize oxygen extraction (8, 14).

Capillary diameter is a key determinant in setting basal flow heterogeneity. The capillary lumen normally range from ~2 to 5 µm in diameter (6). Considering that red blood cells are ~ 6 µm wide (white blood cells being larger) (15), lumen diameter has a significant influence on flow resistance. Capillary diameter is correlated with blood cell velocity and flux, emphasizing its importance in blood flow regulation (6). Pericytes are mural cells that line capillary networks and regulate basal capillary diameter, among many roles in microvascular homeostasis (16). Prior studies have shown that sustained optogenetic stimulation of pericytes can constrict capillaries *in vivo*, and conversely the ablation of pericytes leads to abnormal capillary dilation (6, 17, 18). Capillary pericytes possess some aspects of the contractile machinery expressed by arterial smooth muscle cells (19, 20). However, low to undetectable expression of α-smooth muscle actin (α-SMA) confer slow kinetics that are adequate to support basal capillary flow heterogeneity (6), blood pressure regulation (21), and possibly slower aspects of neurovascular coupling (22). Single cell transcriptomic studies (23) and physiological studies both *in vivo* (24) and *in vitro* (25) also confirm that capillary pericytes express high levels of receptors for vasoconstrictive signaling, such as endothelin 1 type A receptors and thromboxane A2 receptors, potentially involved in basal capillary tone regulation.

While much has been learned about pericyte contributions to capillary tone, prior studies have not examined whether basic structural features of the capillary wall are sufficient to explain heterogeneity in brain capillary diameter. Here, we ask whether the number or thickness of endothelial cells constructing the capillary wall is related to the area and diameter of the capillary lumen. Addressing this question requires broad examination of capillary ultrastructure over the scale of entire capillary networks. With the availability of a new large-scale volume electron microscopy (EM) resource from mouse cerebral cortex called MICrONS Cortical mm^3^ (26, 27), this possibility can now be rigorously tested.

## Methods

### Volume EM data

#### Data collection from Cortical mm^3^

**Figure 1:**
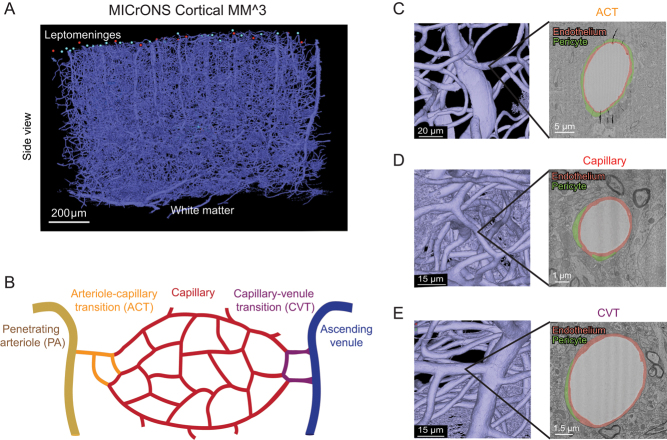
Different vascular zones can be examined within the MICrONS Cortical mm^3^ data set. (A) Entire vascular segmentation within the Cortical MM^3 volume EM data set. (B) Schematic diagram showing different vascular zones as denoted in this study. (C) A penetrating arteriole with branching arteriole–capillary transition (ACT) vessel. A cross section of the ACT vessel is shown, with endothelium and ensheathing pericyte highlighted in orange and green, respectively. The ACT can range from 1 to 4 branch orders from the penetrating arteriole. The average branch order range of 2 is depicted in the schematic. (D) A capillary with the endothelium and pericyte processes highlighted. (E) A capillary–venule transition (CVT) zone vessel (left) and draining ascending venule with the endothelium and pericyte processes highlighted. For this study, we denoted the CVT range as 1–4 branch orders from the ascending venule to match the ACT zone.

Vasculature within the mouse visual cortex was identified in the publicly accessible volume EM resource (Cortical mm^3^) created as part of the IARPA Machine Intelligence from Cortical Network (MICrONS) consortium project (https://www.microns-explorer.org/cortical-mm3). The data set included a 1.4 mm × .87 mm × .84 mm tissue volume from the visual cortex of a P87 mouse. Microvascular data was collected from all 6 cortical layers (and superficial callosal white matter) throughout the data set;* n* = 179 vessels from gray matter and *n* = 6 vessels from white matter. Care was taken not to sample from the same vessel segment twice. Vessels were identified as capillaries when they were branch order ≥ 4 from penetrating arterioles and ascending venules (both denoted as 0 order). Transition zone vessels ranged from branch orders 1 to 4 from penetrating arterioles (ACT) or ascending venules (CVT). The termination of α-SMA, which demarcates the point of transition from ACT to capillary zones, can occur anywhere within the 1–4 branch orders (6, 28). We conservatively used fourth branch order at ACT and CVT as a range to ensure that we accurately targeted the capillary zone, but chose to depict the average branch of α-SMA termination in the ACT zone in Fig. 1B. To confirm vessels were branching from the arteriole side, ensheathing pericytes and perivascular fibroblasts were both required for ACT identity (29). To reduce variation in vessel and endothelial area, images/measurements were taken from areas without endothelial or pericyte nuclei present. This was more challenging within ACT and CVT zones, as nuclei were more abundant in this region (46, 30). However, measurements from ACT zones were sometimes taken when the nuclei of perivascular fibroblasts were present.

#### EM data analysis

Screen captures of identified vessels were taken in Neuroglancer from the 2D EM view of the dataset with scale bars. Images of vessels were imported into and measured using FIJI/ImageJ (NIH), with the scale set according to individual scale bar provided in Neuroglancer. Lumen area/circumference and vessel area/circumference were measured using the freehand selection tool. Lumen diameter of capillary vessels, which were typically circular, was calculated as: SQRT(lumen area/pi). Capillary circularity was calculated as: 4 × pi × A/C^2^, where* A* and *C* are the lumen cross-section area and circumference, respectively. Due to the occasionally elliptical shapes of transition zone vessels, particularly on the venule side, the lumen diameter was measured using the straight-line tool on the minor axis. The endothelial area was calculated from the difference in vessel and lumen area. Endothelial thickness was measured with the straight-line tool at five different points around the vessel and averaged. Percent pericyte coverage was calculated by the length of the vessel circumference in contact with pericyte processes. Source data for EM measurements are provided in the Supplementary Materials.

#### Annotation of endothelial junctions

Annotations were performed in a different volume EM data set collected from layer 2/3 of the visual cortex of a P36 male mouse at 3.58 × 3.58 × 40 nm resolution (31). The vasculature in this dataset consists of capillaries connected to an ascending venule (27, 29). To identify the pattern of endothelial junctions along the capillary vessels, the locations of endothelial junctions were annotation on 2D images every 5–10 slices. 3D images of the raw annotations were then captured to determine the pattern of endothelial–endothelial contact along capillary vessels.

### *In vivo* imaging

#### Mice

*In vivo* deep two-photon imaging data from three adult mice was used for microvascular diameter measurements. The genotypes of these mice were Thy1-YFP (Jax: 003782; one mouse, 24 months old, male) and Atp13a5-2A-CreERT2-IRES-tdTomato (2 mice, 4 months old, male) (32), and all were on a C57Bl/6 background. Room temperature and humidity were maintained within 68–79 °F (setpoint 73 °F) and 30–70% (setpoint 50%), respectively. Mouse chow (LabDiet PicoLab 5053 irradiated diet for standard mice, and LabDiet PicoLab 5058 irradiated diet for breeders) was provided *ad libitum*. The Institutional Animal Care and Use Committee at the Seattle Children’s Research Institute approved all procedures used in this study (protocol #IACUC00396).

#### Surgery

Chronic cranial windows (skull removed, dura intact) were implanted in the skulls of all mice. Briefly, surgical plane anesthesia was induced with a cocktail consisting of fentanyl citrate (0.05 mg/kg), midazolam (5 mg/kg), and dexmedetomidine hydrochloride (0.5 mg/kg) (all from Patterson Veterinary). Dexamethasone (40 µL; Patterson Veterinary) was given 4–6 h prior to surgery to reduce brain swelling during the craniotomy. Circular craniotomies ~4 mm in diameter were generated under sterile conditions and sealed with a glass coverslip consisting of a round 4 mm glass coverslip (Warner Instruments; 64-0724 (CS-4R)) glued to a round 5 mm coverslip (Warner Instruments; 64-0700 (CS-5R)) with UV-cured optical glue (Norland Products; 7110). The coverslip was positioned with the 4 mm side placed directly over the craniotomy, while the 5 mm coverslip laid on the skull surface at the edges of the craniotomy. An instant adhesive (Loctite Instant Adhesive 495) was carefully dispensed along the edge of the 5 mm coverslip to secure it to the skull. Lastly, the area around the cranial window was sealed with dental cement. This two-coverslip ‘plug’ fits precisely into the craniotomy and helps to inhibit skull regrowth, thereby preserving the optical clarity of the window over months. Mice recovered for a minimum of 3 weeks following surgery.

#### Two-photon imaging

*In vivo* two-photon imaging was performed with a Bruker Investigator (run by Prairie View 5.5 software) coupled to a Spectra-Physics Insight X3 laser source. Far red fluorescence emission was collected through a 700/75 nm bandpass filter, respectively, and detected by gallium arsenide phosphide photomultiplier tubes. Low-resolution maps of the cranial window were first collected for navigational purposes using a 4× (0.16 NA) objective (Olympus; UPlanSAPO). We then switched to a 20× (1.0 NA) water-immersion objective (Olympus; XLUMPLFLN) and used 1210 nm excitation to visualize the vasculature using intravenously injected Alexa Fluor 680 dextran, which was custom-conjugated following prior studies (33). All imaging with the water-immersion lens was done with room temperature distilled water. Imaging was performed generally over the primary visual cortex.

#### Quantification of vascular diameter from in vivo data

Capillary and transition zone vascular diameter was measured with the FIJI/ImageJ macro VasoMetrics (33), which provides the average diameter along the vessel segment based on full width at half-maximum fluorescent intensity collected across multiple evenly distributed regions along the vessel length. Multiple measurements along the axis of each capillary also enabled calculation of standard deviation in diameters within an individual capillary segment. As with the volume EM data, vascular metrics were acquired from all six cortical layers (and some callosal white matter) throughout the data set. Source data for *in vivo* measurements are provided in the Supplementary Materials.

#### Statistical analysis

All statistical analysis was performed using GraphPad Prism v9. For all unpaired *t*-tests performed, normality and *F*-tests for variance were performed. When statistically significant *F*-tests were observed, unpaired *t*-test with Welch’s correction for unequal variances was performed. When data with nonnormal Gaussian distributions were observed, nonparametric Mann–Whitney *U* tests were performed. Kruskal–Wallis tests were performed for analysis across CVT, capillary, and CVT zones.

## Results

The MICrONS Cortical mm^3^3 is a volume EM data set encompassing roughly a 1 mm^3^ volume of mouse primary visual cortex. It contains numerous penetrating arterioles and ascending venules, as well as the dense microvascular networks intervening these routes of inflow and outflow (Fig. 1A). We categorized this vasculature into three zones (Fig. 1B): (i) arteriole–capillary transition (ACT), (ii) capillary–venous transition (CVT), and (iii) capillaries (all intervening microvasculature between the ACT and CVT). The ACT zone is defined as a stretch of vasculature spanning between the penetrating arteriole (zeroth order) to the point where expression of α-SMA terminates, which averages 2 branch orders (as depicted in Fig. 1B) but can extend as far as 4 branch orders (6, 34). To achieve a pure sample of capillaries, we used a conservative range of 0 to 4 branch orders from the penetrating arteriole and ascending venule during categorization of ACT and CVT zones, respectively. The vascular lumen segmentation provided in the MICrONS explorer interface was used to navigate through the vascular architecture. We then specifically measured vascular attributes in the ACT, CVT, and capillary zones from 2D images of microvascular crosssections (Fig. 1C, D and E).

**Figure 2:**
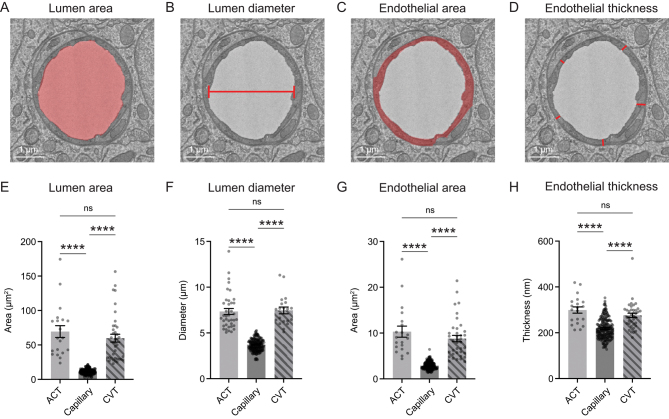
Vessel characteristics across capillary and transitional zones. (A) Measurement of vessel lumen area, as indicated by region of red shading on representative image of capillary. (B) Measurement of lumen diameter. Capillary lumen diameter was extrapolated from the lumen area. For ACT and CVT zones, lumen diameter was the length of the minor axis given their occasional oval shapes. (C) Measurement of endothelial area, as indicated by red shading. (D) Endothelium thickness, as recorded from five locations and averaged per vessel cross section. (E) Comparison of lumen area across microvascular zones. Kruskal–Wallis test: ****P < 0.0001. Dunn’s multiple comparisons test − ACT vs CVT: *P* > 0.99; ACT vs capillary: ****P < 0.0001; capillary vs CVT: ****P < 0.0001. (F) Comparison of lumen diameter across microvascular zones. Kruskal–Wallis test: ****P < 0.0001. Dunn’s multiple comparisons test − ACT vs CVT: *P* > 0.99; ACT vs capillary: ****P < 0.0001; capillary vs CVT: ****P < 0.0001. (G) Comparison of endothelium area across microvascular zones. Kruskal–Wallis test: ****P < 0.0001. Dunn’s multiple comparisons test − ACT vs CVT:, *P* > 0.99; ACT vs capillary: ****P < 0.0001; capillary vs CVT: ****P < 0.0001. (H) Comparison of endothelial thickness across microvascular zones. Kruskal–Wallis test: ****P < 0.0001. Dunn’s multiple comparisons test − ACT vs CVT: *P* > 0.99; ACT vs capillary: ****P < 0.0001; capillary vs CVT ****P < 0.0001. All data shown as mean ± s.e.m.

We quantified the lumen area, lumen diameter, endothelial area, and endothelial thickness of individual vessels across these three microvascular zones (Fig. 2A, B, C, and D). Microvessels were sampled across all cortical layers (with some sampling in superficial corpus callosum) and then pooled to gain an overall view of their characteristics. A broad range of diameters were measured in each microvascular zone. Lumen area and diameter of ACT and CVT zones were larger than those of capillaries (Fig. 2A, B, E, and F). As expected, the area of the endothelium was greater with larger diameter vessels (Fig. 2C and G). Endothelial thickness was significantly larger in ACT and CVT zones, compared to capillaries (Fig. 2D and H).

**Figure 3:**
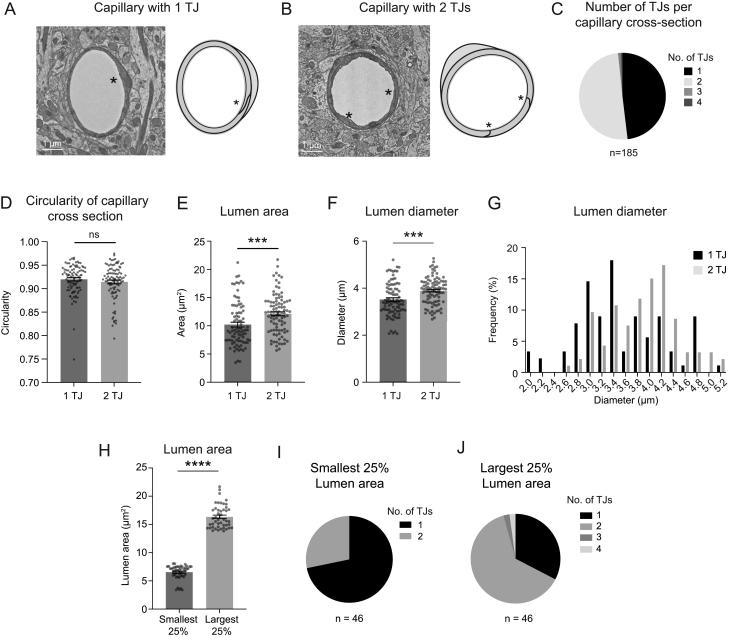
Influence of endothelial cell number on capillary size. (A) Representative image and schematic of capillary with one tight junction (one TJ represented with *), and therefore one endothelial cell. (B) Representative image and schematic of capillary with two tight junctions (two TJs represented with *, one each), and therefore two interlocked endothelial cells in the vessel wall. (C) Distribution of TJ number across 185 capillaries. Our sampled group had 89 (48.11%) capillaries with one TJ, and 93 (50.27%) capillaries with two TJs. Only two capillaries (1.08% of total) were found with three TJs and 1 capillary (0.54% of total) found with four TJs. (D) Circularity of capillary cross sections between one TJ and two TJ groups was not different. Mann–Whitney *U* test; *P* = 0.4432. (E) Comparison of lumen area between capillaries with one and two TJs. Unpaired *t*-test (two-sided), *t*(178) = 3.357; ***P = 0.0010. *n* = 89 capillaries with one TJ, *n* = 93 capillaries with two TJs. Data shown as mean ± s.e.m. (F) Comparison of lumen diameter between capillaries with one and two TJs. Unpaired *t*-test (two-sided), *t*(170.3) = 3.854; ***P = 0.0002. *n* = 89 capillaries with one TJ, *n* = 93 capillaries with two TJs. Data shown as mean ± s.e.m . (G) Frequency distribution of lumen diameter of capillaries with one and two TJs. (H) Lumen area for smallest and largest capillaries. Unpaired *t*-test with Welch’s correction (two-sided), *t*(73.66) = 27.49; ****P < 0.0001. (I, J) Distribution of TJ number in the smallest and largest capillaries.

To determine whether capillary diameter heterogeneity was influenced by the number of endothelial cells in the vessel wall, each capillary cross section was examined for tight junction (TJ) number by three independent raters (authors: SMS, SKB, VCS). A capillary with a single TJ is composed of a single endothelial cell wrapping and connecting with itself (Fig. 3A). A capillary with two TJs is composed of two endothelial cells interlocking to form the vessel wall (Fig. 3B). The capillary zone contained predominantly capillaries with one or two TJs, with roughly equal proportions (Fig. 3C). Capillaries with three and four TJs, indicating three and four endothelial cells respectively, were also observed on rare occasions (3 out of 185 capillaries examined). In contrast, these multi-TJ vessels were common in the ACT and CVT zones, with up to five or six TJs per vessel observed (Supplementary Fig. 1, see section on supplementary materials given at the end of this article).

Capillary cross sections may not be cut exactly perpendicular to the longitudinal axis of the capillary, which can affect quantifications of lumen size. We calculated the circularity of capillary cross sections in both one and two TJ groups and detected no difference, which ensured there was no bias in capillary sample collection between groups (Fig. 3D). We compared vascular attributes between capillaries with one or two TJs. Lumen area and diameter were on average significantly larger with two TJ capillaries. The average capillary lumen areas and diameter were 2 μm^2^ and 0.4 μm larger with two TJs in comparison to a single TJ, respectively (Fig. 3E and F). The range in capillary lumen area (~3–21 μm^2^) and diameter (~2–5 μm) was broad and overlapped heavily between the two groups (Fig. 3G). Interestingly, capillaries 3.4 μm and smaller tended to be composed of one endothelial cell, while those 3.6 μm and larger tended to be composed of two endothelial cells.

As a further strategy to verify the influence of endothelial cell number on capillary lumen area, we examined the upper and lower extremes of lumen area within the capillary group. We separated the smallest 25% of lumen areas (lower) and the largest 25% of lumen areas (upper) and compared the distribution of endothelial TJs numbers in these groups (Fig. 3H). Vessels in the lower 25% group were composed mostly of those with one TJ, while the upper 25% were predominantly vessels with two TJs, and contained the rare vessels with three and four TJs (Fig. 3I and J). This again shows that larger diameter capillaries are likely to be composed of two or more endothelial cells. Overall, these data suggest that endothelial number contributes to capillary diameter, but alone is insufficient to explain the full range of capillary diameters observed.

We also considered the possibility that a single TJ strands could pass the capillary cross section multiple times, leading to overestimation of endothelial cell number. Examination of TJ strands orientation using annotations in Neuroglancer, as previously described (35), revealed that endothelial cells are typically elongated in the longitudinal axis of the capillary, making it unlikely that TJ strands to meander in and out of a single cross-sectional plane (Supplementary Fig. 2). However, this could not be examined for all capillaries and is a limitation of our analysis procedure.

**Figure 4:**
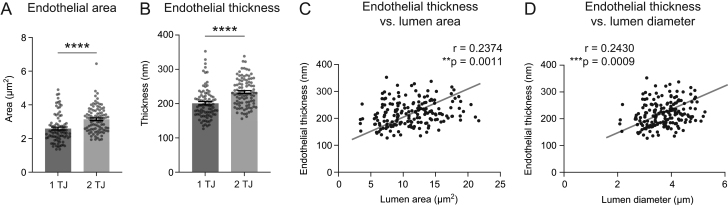
Relationship between capillary area and endothelial area or thickness. (A) Comparison of endothelial area between one TJ and two TJ capillaries. Unpaired *t*-test (two-sided), *t*(180) = 4.773; ****P < 0.0001. *n* = 89 capillaries with one TJ, *n* = 93 capillaries with two TJ. (B) Comparison of endothelial thickness between one TJ and two TJ capillaries. Unpaired *t*-test (two-sided), *t*(180) = 5.076; ****P < 0.0001. *n* = 89 capillaries with one TJ, *n* = 93 capillaries with two TJs. Data shown as mean ± s.e.m. Data shown as mean ± s.e.m. (C) Endothelial thickness plotted as a function of lumen area. **P = 0.0011 (two-sided). Pearson *r* = 0.2374. *R^2^* = 0.05637. (D) Endothelial thickness plotted as a function of lumen diameter. ***P = 0.0009 (two-sided). Pearson* r* = 0.2430. *R*^2^ = 0.05903.

We next examined how other attributes of the endothelium related to lumen size. As expected, the area of the endothelial cross section was greater with the larger lumen areas of two TJ capillaries (Fig. 4A). We considered if distention of endothelium was necessary to create larger diameter capillaries, i.e. whether larger capillaries have thinner walls due to cell stretching. Instead, we found that larger capillaries exhibited thicker endothelial walls, and that there was an overall positive relationship between lumen area/diameter and endothelial thickness (Fig. 4B, C and D). Lending confidence to the accuracy of our measurements, values for capillary lumen area and endothelial thickness are concordant with those measured in prior studies (36).

We further asked whether heterogeneity in capillary diameter was related to the extent of pericyte coverage at the cross sections examined. Pericyte coverage was measured as the percentage of capillary wall contacted by pericyte processes in each image (Supplementary Fig. 3). This analysis revealed no difference in pericyte coverage between one and two TJ capillaries. We also found no correlation between pericyte coverage and other attributes of the capillaries examined (Supplementary Fig. 4). Critically, we note that pericyte coverage can differ substantially based on location along a capillary segment, such as proximity to the pericyte soma (29), and future studies will need to reexamine the influence of pericyte coverage using 3D analysis. To summarize these findings alongside endothelial attributes mentioned earlier, we constructed a Pearson’s correlation matrix on metrics extracted from the volume EM data (Supplementary Fig. 4).

**Figure 5:**
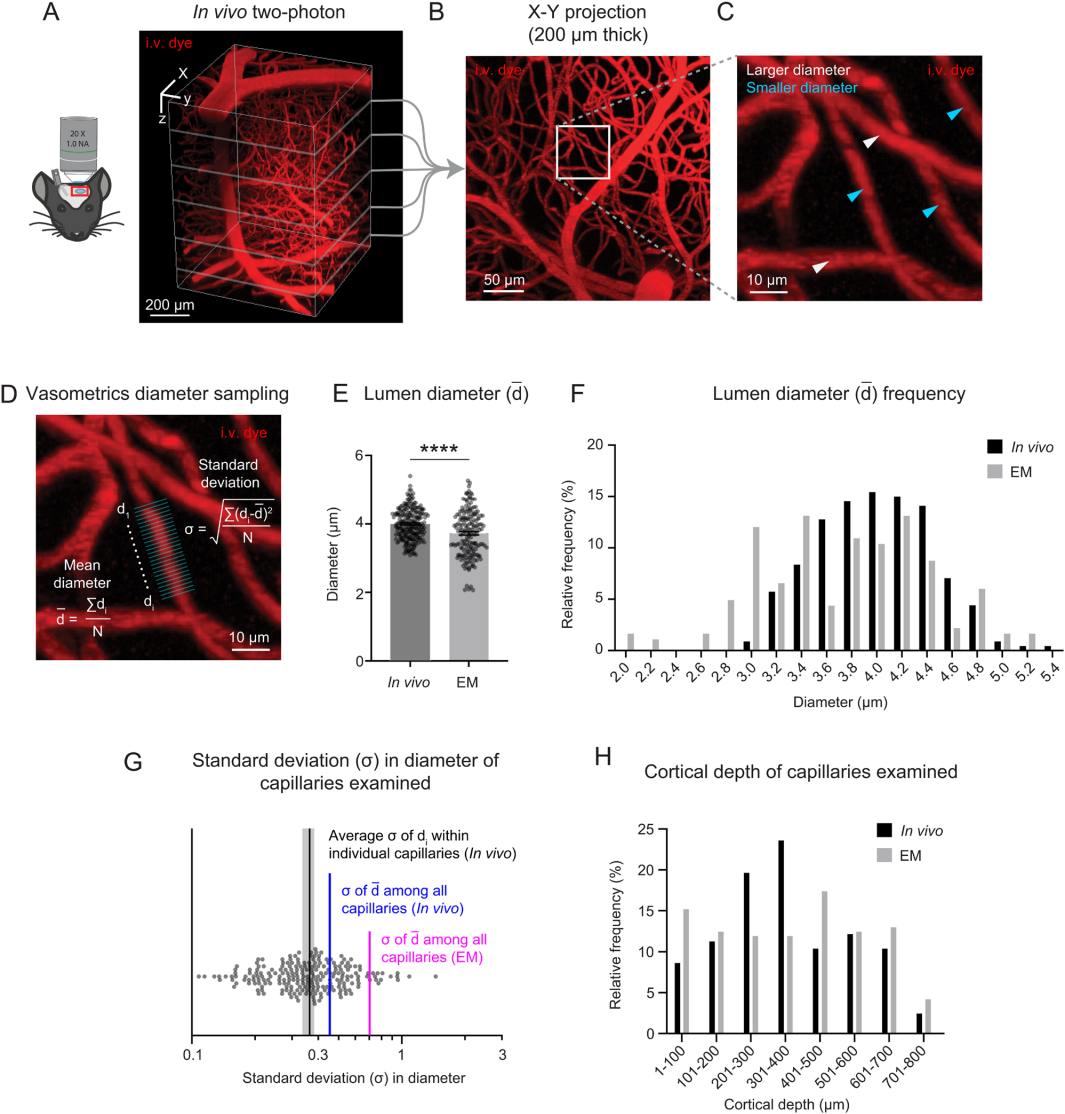
Volume EM data exhibits slightly smaller average capillary diameter than seen* in vivo* but retains heterogeneity in capillary diameter. (A) Deep* in vivo* two-photon imaging of isoflurane anesthetized mice via cranial window using Alexa Fluor 680 dextran (i.v. dye). 3D rendering of microvasculature within the mouse primary visual cortex. (B) Maximal projection from 250 to 450 μm of cortical depth. (C) Inset shows example region of diameter measurement for individual capillaries, with white arrows showing larger capillaries and cyan arrows showing smaller capillaries. (D) Vasometrics diameter sampling measured capillary diameter at multiple locations along the longitudinal axis of the capillary. Equations are shown for mean diameter and standard deviation of diameter within a capillary segment. (E) Comparison of capillary lumen diameters between *in vivo* two-photon imaging and volume EM data. Unpaired *t*-test with Welch's correction (two-sided), *t*(307.9) = 4.670; ****P < 0.0001. *n* = 227 capillaries from three adult mice for *in vivo* data; *n* = 183 capillaries from 1 mouse for the MICrONS Cortical mm^3^ data. Data shown as mean ± s.e.m.(F) Frequency distribution of lumen diameters from each data type. Capillary diameters were measured across all cortical layers. (G) Scatter plot showing standard deviation of diameters measured within individual capillary segments, with standard deviation of diameters among capillaries measured *in vivo* and in EM data. (H) Cortical depths of capillaries sampled *in vivo* and in EM data.

Finally, we asked whether tissue fixation and processing could affect capillary diameter ranges in the Cortical mm^3^ data. Insufficient intravascular pressure during transcranial perfusion and fixation procedures could conceivably lead to collapsed or altered vascular lumen within volume EM data. To collect ground truth data, *in vivo* deep two-photon imaging was performed in the visual cortex of three adult mice under isoflurane anesthesia to measure capillary diameters from the pial surface to the gray and white matter interface (Fig. 5A, B, C and D). Our prior studies showed that isoflurane does not lead to dilation of capillaries compared to lightly sedated or awake mice (6, 37). We found that the diameter of capillaries in Cortical mm^3^ were, on average, slightly smaller in diameter than that seen *in vivo* (Fig. 5E). However, the range of capillary diameters (~3–5 μm) was similar between* in vivo* and volume EM data (Fig. 5F). This confirms that perfusion fixation and tissue handling used to generate the Cortical mm^3^ data preserved the expected range in capillary diameter typically seen *in vivo*.

We next asked how variance within individual capillary segments compared to variance among capillaries within a network. Shifts in endothelial cell number, or occurrence of pericyte somata, could alter capillary diameter (38). Since *in vivo* capillaries were measured at multiple locations along their length using our analysis software, Vasometrics (39), this analysis could be conducted. Lumen diameter measurements were taken at 1 μm increments for an average length of 26.96 μm ± 12.77 μm (mean ± s.d.) along each capillary segment; the median length of microvessels in mouse cortex is 50 μm (40). The average of standard deviations within individual capillaries (0.391 ± 0.02; average ± 95% CI) was smaller than standard deviations of diameters among all capillaries sampled (0.454) (Fig. 5G). This indicates that heterogeneity in capillary diameter is driven more by variation between capillary segments than within segments, although the latter did have some contribution. As expected from existence of some very small diameter capillaries in the EM data, the standard deviation of capillary diameters (0.704) was higher than that measured *in vivo*.

Finally, since capillary diameter and heterogeneity may differ based on cortical layer, we show that capillaries were sampled over a similar range of cortical depths between the *in vivo* and EM data. This provides confidence that similar types of capillaries were compared across animals (Fig. 5H).

## Discussion

In this study, we used large-scale volume EM data (26) to show that endothelial cell number has a partial influence on microvascular lumen diameter and area. Microvessels in transitional zones near penetrating arterioles and ascending venules are larger and typically composed of 2 to 6 endothelial cells, while capillaries intervening these regions are constructed from either one or two endothelial cells at a nearly 50 : 50 ratio. We show that capillaries with two endothelial cells are, on average, larger than those with one endothelial cell. However, the diameter ranges of capillaries with one and two endothelial cells are broad and overlapping, suggesting that endothelial cell number cannot explain the full range of capillary diameters observed *in vivo*. Finally, we use deep two-photon imaging to verify that capillary diameter ranges captured in volume EM are comparable to that measured *in vivo*.

Microvascular architecture is established during cerebrovascular development (41), and shaped by blood flow (42) and the metabolic demands (43) of the growing brain. Whether endothelial structure is stable in the adult brain or actively remodeled has not been deeply examined. Some studies have tracked endothelial cells (and pericytes) longitudinally using *in vivo* two-photon imaging and showed relatively stable endothelial cell position and tight junction arrangement during adulthood, at least over the timeframe of days to weeks (44, 45). Cudmore *et al.* used a Tie2-based Cre driver to track capillaries in the motor cortex over time while mice had access to a running wheel (38). Interestingly, they reported marked stability of pericytes and endothelial cell density, except for small occasional shifts in nuclei position along the vessel wall. Reeson *et al.* showed that endothelial cells can be induced to reposition in the adult brain during local regression events caused by microvascular occlusions, indicating the potential to remodel in response to pathological stimuli (11). Endothelial cells that regress, migrate to nearby vessel segments and therefore increase endothelial cell number. Thus, endothelial contributions to capillary flow heterogeneity must be established during cerebrovascular development. Endothelial structure is stable in the adult cerebral cortex, but remodeling can be evoked with flow capillary obstruction. More longitudinal imaging studies are needed to determine how this process unfolds during development (46), and whether long-term alterations in neural activity can reshape capillary flow heterogeneity in development and adulthood (37, 47).

Our findings support the existence of additional mechanisms beyond endothelial number that control capillary diameter. As discussed earlier, capillary tone provided by capillary pericytes is a logical mechanism. There is now ample evidence for the contractile ability of capillary pericytes (6, 17, 18, 28), but the endogenous vasoconstrictive signals that create this tone under basal conditions remain to be determined. Unraveling this mechanism will require experiments to conditionally delete receptors for vasoconstrictive signals known to be used by pericytes such as endothelin-1, thromboxane A2, and noradrenaline receptors. Another potential contributor that remains poorly understood is tone generation from the cytoskeletal elements of the endothelial cells themselves, which is far less studied than pericyte contractility (48, 49). Further structural attributes of the vessel wall could include endothelial nuclei, which can protrude into the luminal space or the prevalence of small finger-like protrusions in the lumen called endothelial microvilli, which could both lead to increased local flow resistance (27).

There are some limitations to our study. First, the ultrastructural data is derived from the primary visual cortex of a single mouse. As volume EM data becomes more readily available, it will be possible to reexamine our hypothesis more broadly. Second, despite being able to sample hundreds of capillaries within Cortical mm^3^, we only quantified ones cut perpendicular to the plane of highest spatial resolution, and therefore introduced bias toward a subset of capillaries oriented in one plane. Third, we detected some variance in diameter within capillary segments, but our analyses relied on 2D cross sections in restricted regions. Deeper investigations on the basis of this variation, be it shifts in endothelial cell number, presence of pericyte somata (32) or other factors, will require 3D reconstructions and additional proofreading efforts to rigorously segment pericyte and endothelial compartments. Fourth, the issue of how tissue fixation and handling affect the native structure of the vascular lumen and wall components requires deeper investigation. Fixation approaches have a strong influence when preserving the extracellular space during EM (50, 51). By comparing variance on capillary diameter between Cortical mm^3^ and data collected from anesthetized mice using *in vivo* deep two-photon imaging, we see a similar range of capillary diameters, which lends confidence to the idea that capillary diameters were generally preserved.

In the Alzheimer’s brain, and in many related neurological diseases, capillary diameter is altered, and microvascular density is reduced. This is expected to impair blood flow by increasing flow resistance but will also disrupt the range of capillary diameters that is critical for blood distribution. Further, shifts toward increased basal capillary heterogeneity may raise the threshold to distribute blood and oxygen during functional hyperemia. How alterations in endothelial structure and density factor into these disease-related microvascular deficits remains heavily understudied, yet vital to understanding mechanistic targets for improvement of microvascular perfusion.

## Supplementary Materials

Supplementary Figures

Supplementary Table 1

Supplementary Table 2

Supplementary Table 3

## Declaration of interest

The authors declare that there is no conflict of interest that could be perceived as prejudicing the impartiality of the study reported.

## Funding

Our work is supported by grants to AS from the NIH/NINDS (NS097775) and NIH/NIA (AG062738, R21AG069375, RF1AG077731). SKB was supported by a fellowship from the National Institutes of Health, National Institutes of Neurological Disease and Stroke (F32NS117649). VCS was supported by a junior leader fellowship from ‘La Caixa’ Foundation (LCF/BQ/PI22/11910036).

## Author contribution statement

This project was conceived by AYS, and all analyses were performed by SMS. SKB and VCS served as independent raters of tight junction number analysis. YL and SS contributed *in vivo* deep imaging data sets. MT provided consultation on the MICrONS data set. The manuscript was written by AYS with feedback from all authors.

## References

[1] Seylaz J Charbonne R Nanri K Von Euw D Borredon J Kacem K Meric P & Pinard E. Dynamic *in vivo* measurement of erythrocyte velocity and flow in capillaries and of microvessel diameter in the rat brain by confocal laser microscopy. Journal of Cerebral Blood Flow and Metabolism 1999 19 863–870. (10.1097/00004647-199908000-00005) 10458593

[2] Kleinfeld D Mitra PP Helmchen F & Denk W. Fluctuations and stimulus-induced changes in blood flow observed in individual capillaries in layers 2 through 4 of rat neocortex. PNAS 1998 95 15741–15746. (10.1073/pnas.95.26.15741) 9861040 PMC28114

[3] Dirnagl U Villringer A & Einhaupl KM. *In-vivo* confocal scanning laser microscopy of the cerebral microcirculation. Journal of Microscopy 1992 165 147–157. (10.1111/j.1365-2818.1992.tb04312.x) 1552568

[4] Villringer A Them A Lindauer U Einhaupl K & Dirnagl U. Capillary perfusion of the rat brain cortex: an *in vivo* confocal microscopy study. Circulation Research 1994 75 55–62. (10.1161/01.res.75.1.55) 8013082

[5] Moeini M Lu X Avti PK Damseh R Bélanger S Picard F Boas D Kakkar A & Lesage F. Compromised microvascular oxygen delivery increases brain tissue vulnerability with age. Scientific Reports 2018 8 8219. (10.1038/s41598-018-26543-w) 29844478 PMC5974237

[6] Hartmann DA Berthiaume AA Grant RI Harrill SA Koski T Tieu T McDowell KP Faino AV Kelly AL & Shih AY. Brain capillary pericytes exert a substantial but slow influence on blood flow. Nature Neuroscience 2021 24 633–645. (10.1038/s41593-020-00793-2) 33603231 PMC8102366

[7] Drew PJ Shih AY & Kleinfeld D. Fluctuating and sensory-induced vasodynamics in rodent cortex extends arteriole capacity. Proceedings of the National Academy of Sciences of the United States of America 2011 108 8473–8478. (10.1073/pnas.1100428108) 21536897 PMC3100929

[8] Jespersen SN & Østergaard L. The roles of cerebral blood flow, capillary transit time heterogeneity, and oxygen tension in brain oxygenation and metabolism. Journal of Cerebral Blood Flow and Metabolism 2012 32 264–277. (10.1038/jcbfm.2011.153) 22044867 PMC3272609

[9] Poole DC Copp SW Ferguson SK & Musch TI. Skeletal muscle capillary function: contemporary observations and novel hypotheses. Experimental Physiology 2013 98 1645–1658. (10.1113/expphysiol.2013.073874) 23995101 PMC4251469

[10] Lam CK Yoo T Hiner B Liu Z & Grutzendler J. Embolus extravasation is an alternative mechanism for cerebral microvascular recanalization. Nature 2010 465 478–482. (10.1038/nature09001) 20505729 PMC2879083

[11] Reeson P Choi K & Brown CE. VEGF signaling regulates the fate of obstructed capillaries in mouse cortex. eLife 2018 7 e33670. (10.7554/eLife.33670) 29697373 PMC5919759

[12] Li Y Wei W & Wang RK. Capillary flow homogenization during functional activation revealed by optical coherence tomography angiography based capillary velocimetry. Scientific Reports 2018 8 4107. (10.1038/s41598-018-22513-4) 29515156 PMC5841298

[13] Li B Lee J Boas DA & Lesage F. Contribution of low- and high-flux capillaries to slow hemodynamic fluctuations in the cerebral cortex of mice. Journal of Cerebral Blood Flow and Metabolism 2016 36 1351–1356. (10.1177/0271678X16649195) 27165011 PMC4976754

[14] Li B Esipova TV Sencan I Kılıç K Fu B Desjardins M Moeini M Kura S Yaseen MA Lesage F, More homogeneous capillary flow and oxygenation in deeper cortical layers correlate with increased oxygen extraction. eLife 2019 8 e42299. (10.7554/eLife.42299) 31305237 PMC6636997

[15] Fukuda T Asou E Nogi K & Goto K. Evaluation of mouse red blood cell and platelet counting with an automated hematology analyzer. Journal of Veterinary Medical Science 2017 79 1707–1711. (10.1292/jvms.17-0387) 28845024 PMC5658564

[16] Sweeney MD Ayyadurai S & Zlokovic BV. Pericytes of the neurovascular unit: key functions and signaling pathways. Nature Neuroscience 2016 19 771–783. (10.1038/nn.4288) 27227366 PMC5745011

[17] Nelson AR Sagare MA Wang Y Kisler K Zhao Z & Zlokovic BV. Channelrhodopsin excitation contracts brain pericytes and reduces blood flow in the aging mouse brain in vivo. Frontiers in Aging Neuroscience 2020 12 108. (10.3389/fnagi.2020.00108) 32410982 PMC7201096

[18] O'Herron PJ Hartmann DA Xie K Kara P & Shih AY. 3D optogenetic control of arteriole diameter in vivo. eLife 2022 11 e72802. (10.7554/eLife.72802) 36107146 PMC9481242

[19] Alarcon-Martinez L Yilmaz-Ozcan S Yemisci M Schallek J Kılıç K Can A Di Polo A & Dalkara T. Capillary pericytes express α-smooth muscle actin, which requires prevention of filamentous-actin depolymerization for detection. eLife 2018 7 e34861. (10.7554/eLife.34861) 29561727 PMC5862523

[20] Gonzales AL Klug NR Moshkforoush A Lee JC Lee FK Shui B Tsoukias NM Kotlikoff MI Hill-Eubanks D & Nelson MT. Contractile pericytes determine the direction of blood flow at capillary junctions. PNAS 2020 117 27022–27033. (10.1073/pnas.1922755117) 33051294 PMC7604512

[21] Klug NR Sancho M Gonzales AL Heppner TJ O'Brien RIC Hill-Eubanks D & Nelson MT. Intraluminal pressure elevates intracellular calcium and contracts CNS pericytes: role of voltage-dependent calcium channels. PNAS 2023 120 e2216421120. (10.1073/pnas.2216421120) 36802432 PMC9992766

[22] Rungta RL Chaigneau E Osmanski BF & Charpak S. Vascular compartmentalization of functional hyperemia from the synapse to the pia. Neuron 2018 99 362–375.e4. (10.1016/j.neuron.2018.06.012) 29937277 PMC6069674

[23] Vanlandewijck M He L Mäe MA Andrae J Ando K Del Gaudio F Nahar K Lebouvier T Laviña B Gouveia L, A molecular atlas of cell types and zonation in the brain vasculature. Nature 2018 554 475–480. (10.1038/nature25739) 29443965

[24] Fernández-Klett F Offenhauser N Dirnagl U Priller J & Lindauer U. Pericytes in capillaries are contractile *in vivo*, but arterioles mediate functional hyperemia in the mouse brain. PNAS 2010 107 22290–22295. (10.1073/pnas.1011321108) 21135230 PMC3009761

[25] Nortley R Korte N Izquierdo P Hirunpattarasilp C Mishra A Jaunmuktane Z Kyrargyri V Pfeiffer T Khennouf L Madry C, Amyloid β oligomers constrict human capillaries in Alzheimer's disease via signaling to pericytes. Science 2019 365. (10.1126/science.aav9518) PMC6658218 31221773

[26] The MICrONS Consortium Bae JA Baptiste M Bodor AL Brittain D Buchanan J Bumbarger DJ Castro MA Celii B Cobos E, Functional connectomics spanning multiple areas of mouse visual cortex. bioRxiv 2021. (10.1101/2021.07.28.454025) PMC11981939 40205214

[27] Bonney SK Coelho-Santos V Huang SF Takeno M Kornfeld J Keller A & Shih AY. Public volume electron microscopy data: an essential resource to study the brain microvasculature. Frontiers in Cell and Developmental Biology 2022 10 849469. (10.3389/fcell.2022.849469) 35450291 PMC9016339

[28] Grant RI Hartmann DA Underly RG Berthiaume AA Bhat NR & Shih AY. Organizational hierarchy and structural diversity of microvascular pericytes in adult mouse cortex. Journal of Cerebral Blood Flow and Metabolism 2019 39 411–425. (10.1177/0271678X17732229) 28933255 PMC6399730

[29] Bonney SK Sullivan LT Cherry TJ Daneman R & Shih AY. Distinct features of brain perivascular fibroblasts and mural cells revealed by in vivo two-photon imaging. Journal of Cerebral Blood Flow and Metabolism 2022 42 966–978. (10.1177/0271678X211068528) 34929105 PMC9125487

[30] Shaw K Boyd K Anderle S Hammond-Haley M Amin D Bonnar O & Hall CN. Gradual not sudden change: multiple sites of functional transition across the microvascular bed. Frontiers in Aging Neuroscience 2021 13 779823. (10.3389/fnagi.2021.779823) 35237142 PMC8885127

[31] Schneider-Mizell CM Bodor AL Collman F Brittain D Bleckert A Dorkenwald S Turner NL Macrina T Lee K Lu R, Structure and function of axo-axonic inhibition. eLife 2021 10 e73783. (10.7554/eLife.73783) 34851292 PMC8758143

[32] Guo X Ge T Xia S Wu H Colt M Xie X Zhang B Zeng J Chen J Zhu D, Atp13a5 marker reveals pericytes of the central nervous system in mice. bioRxiv 2021. (10.1101/2021.07.09.451694)

[33] Li B Ohtomo R Thunemann M Adams SR Yang J Fu B Yaseen MA Ran C Polimeni JR Boas DA, Two-photon microscopic imaging of capillary red blood cell flux in mouse brain reveals vulnerability of cerebral white matter to hypoperfusion. Journal of Cerebral Blood Flow and Metabolism 2020 40 501–512. (10.1177/0271678X19831016) 30829101 PMC7026840

[34] Hartmann DA Coelho-Santos V & Shih AY. Pericyte control of blood flow across microvascular zones in the central nervous system. Annual Review of Physiology 2022 84 331–354. (10.1146/annurev-physiol-061121-040127) PMC10480047 34672718

[35] Ornelas S Berthiaume AA Bonney SK Coelho-Santos V Underly RG Kremer A Guérin CJ Lippens S & Shih AY. Three-dimensional ultrastructure of the brain pericyte-endothelial interface. Journal of Cerebral Blood Flow and Metabolism 2021 41 2185–2200. (10.1177/0271678X211012836) 33970018 PMC8393306

[36] Nahirney PC Reeson P & Brown CE. Ultrastructural analysis of blood-brain barrier breakdown in the peri-infarct zone in young and aged mice. Journal of Cerebral Blood Flow and Metabolism 2015 36 413–425. (10.1177/0271678X1560839) 26661190 PMC4759675

[37] Berthiaume AA Schmid F Stamenkovic S Coelho-Santos V Nielson CD Weber B Majesky MW & Shih AY. Pericyte remodeling is deficient in the aged brain and contributes to impaired capillary flow and structure. Nature Communications 2022 13 5912. (10.1038/s41467-022-33464-w) PMC9547063 36207315

[38] Davis H & Attwell D. A tight squeeze: how do we make sense of small changes in microvascular diameter? Journal of Physiology 2023 601 2263–2272. (10.1113/JP284207) 37036208 PMC10953087

[39] McDowell KP Berthiaume AA Tieu T Hartmann DA & Shih AY. VasoMetrics: unbiased spatiotemporal analysis of microvascular diameter in multi-photon imaging applications. Quantitative Imaging in Medicine and Surgery 2021 11 969–982. (10.21037/qims-20-920) 33654670 PMC7829163

[40] Blinder P Tsai PS Kaufhold JP Knutsen PM Suhl H & Kleinfeld D. The cortical angiome: an interconnected vascular network with noncolumnar patterns of blood flow. Nature Neuroscience 2013 16 889–897. (10.1038/nn.3426) 23749145 PMC4141079

[41] Coelho-Santos V & Shih AY. Postnatal development of cerebrovascular structure and the neurogliovascular unit. Wiley Interdisciplinary Reviews. Developmental Biology 2020 9 e363. (10.1002/wdev.363) 31576670 PMC7027551

[42] Chen Q Jiang L Li C Hu D Bu JW Cai D & Du JL. Haemodynamics-driven developmental pruning of brain vasculature in zebrafish. PLOS Biology 2012 10 e1001374. (10.1371/journal.pbio.1001374) 22904685 PMC3419171

[43] Lacoste B Comin CH Ben-Zvi A Kaeser PS Xu X Costa Lda F & Gu C. Sensory-related neural activity regulates the structure of vascular networks in the cerebral cortex. Neuron 2014 83 1117–1130. (10.1016/j.neuron.2014.07.034) 25155955 PMC4166422

[44] Cudmore RH Dougherty SE & Linden DJ. Cerebral vascular structure in the motor cortex of adult mice is stable and is not altered by voluntary exercise. Journal of Cerebral Blood Flow and Metabolism 2017 37 3725–3743. (10.1177/0271678X16682508) 28059584 PMC5718320

[45] Murphy PA Kim TN Huang L Nielsen CM Lawton MT Adams RH Schaffer CB & Wang RA. Constitutively active Notch4 receptor elicits brain arteriovenous malformations through enlargement of capillary-like vessels. PNAS 2014 111 18007–18012. (10.1073/pnas.1415316111) 25468970 PMC4273347

[46] Coelho-Santos V Berthiaume AA Ornelas S Stuhlmann H & Shih AY. Imaging the construction of capillary networks in the neonatal mouse brain. PNAS 2021 118 e2100866118. (10.1073/pnas.2100866118) 34172585 PMC8256089

[47] Whiteus C Freitas C & Grutzendler J. Perturbed neural activity disrupts cerebral angiogenesis during a postnatal critical period. Nature 2014 505 407–411. (10.1038/nature12821) 24305053 PMC3947100

[48] Erdener ŞE Küreli G & Dalkara T. Contractile apparatus in CNS capillary pericytes. Neurophotonics 2022 9 021904. (10.1117/1.NPh.9.2.021904) 35106320 PMC8785978

[49] Kureli G Yilmaz-Ozcan S Erdener SE Donmez-Demir B Yemisci M Karatas H & Dalkara T. F-actin polymerization contributes to pericyte contractility in retinal capillaries. Experimental Neurology 2020 332 113392. (10.1016/j.expneurol.2020.113392) 32610106

[50] Cragg B. Preservation of extracellular space during fixation of the brain for electron microscopy. Tissue and Cell 1980 12 63–72. (10.1016/0040-8166(8090052-x) 6987773

[51] Pallotto M Watkins PV Fubara B Singer JH & Briggman KL. Extracellular space preservation aids the connectomic analysis of neural circuits. eLife 2015 4 e08206. (10.7554/eLife.08206) 26650352 PMC4764589

