# Supplementary material for: Endothelial structure contributes to heterogeneity in brain capillary diameter
Source: Vasc Biol. 2023 Sep 6;5(1):e230010. doi: 10.1530/VB-23-0010 (PMC10503221; doi:10.1530/VB-23-0010)
Supplement: Supplementary Figures [file supplementary_figures.pdf]

Supplementary Figures:

Endothelial structure contributes to heterogeneity in brain capillary diameter

By Sheridan M. Sargent, Stephanie K. Bonney, Yuandong Li, Stefan Stamenkovic, Marc Takeno, Vanessa Coelho-Santos, and Andy Y. Shih

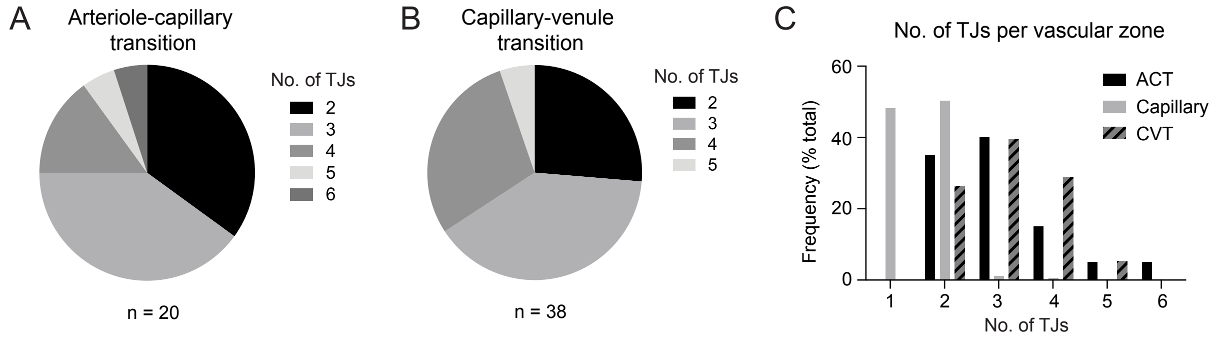

Supplementary Figure 1. Tight junction number in transitional zones. **(A,B)** Distribution of TJ number in ACT (A) and CVT (B) zones. **(C)** Frequency distribution of TJ number across ACT, capillary and CVT zones.

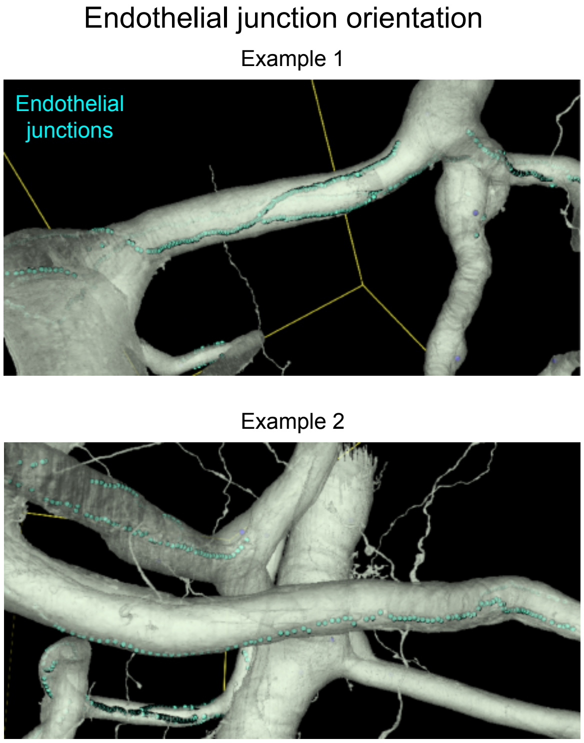

Supplementary Figure 2. Endothelial tight junctions orient longitudinally along the length of brain capillaries.The location of endothelial tight junctions were annotated in Neuroglancer and 3D renderings were created for two examples.Adapted from Ornelas et al. JCFBFM, 2021 (PMID: 33970018).

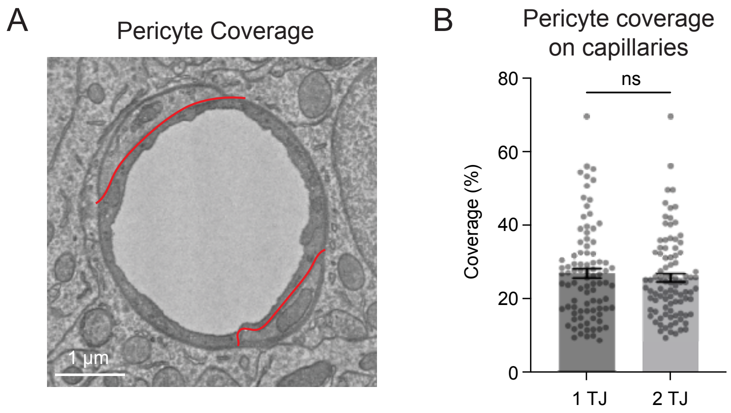

Supplementary Figure 3. Pericyte coverage between 1 and 2 TJ capillaries. **(A)** Example of capillary cross section with red lines marking the pericyte-endothelial interface. **(B)** Pericyte coverage between 1 TJ and 2 TJ capillaries. Unpaired t test (two-sided), $t(180)=0.6902; p=0.4909$. N=89 capillaries with 1 TJ, n=93 capillaries with 2 TJ. Data shown as mean ± SEM.

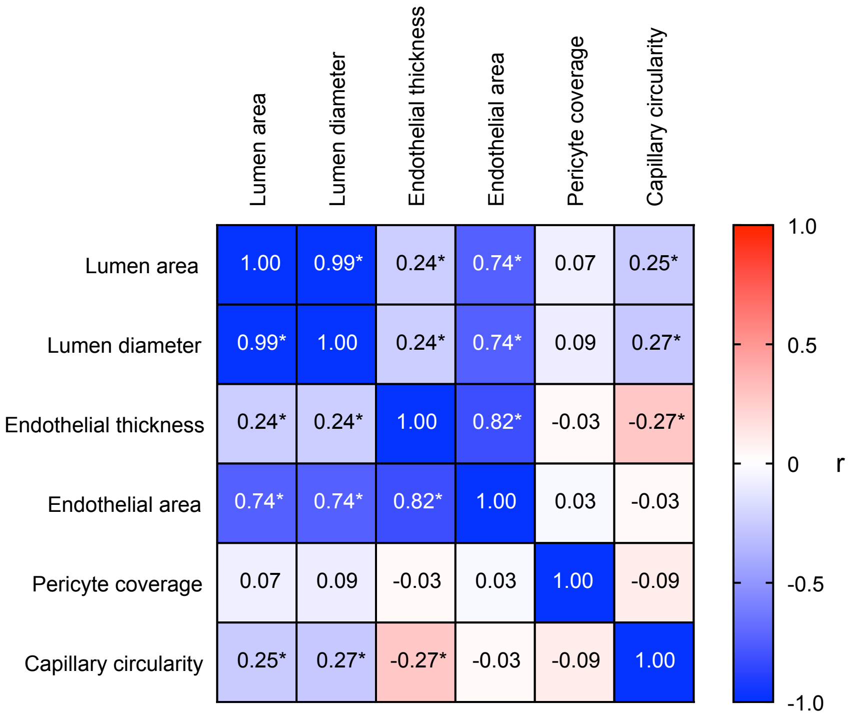

Supplementary Figure 4. Pearsons correlation matrix for capillary metrics measured from volume EM data. The matrix is derived from n=185 capillaries. The depth of the color represents the strength of the correlation, and the colors blue or red represent positive or negative correlations, respectively. Correlations with statistical signficance of $p \leq 0.001$ are denoted with an asterisk.
